# Synthetic data for more accurate deep learning models in molecular science: a test case of protein-ligand binding affinity prediction

**DOI:** 10.1186/s13321-026-01279-2

**Published:** 2026-08-13

**Authors:** Prakul Agrawal, Prathit Chatterjee, U. Deva Priyakumar

**Affiliations:** Center for Computational Natural Sciences and Bioinformatics, International Institute of Information Technology, Hyderabad, 500032 India

**Keywords:** Synthetic data, Binding affinity, Deep learning, Data augmentation

## Abstract

Deep learning models are data-hungry, and synthetic (artificial) data has been shown to be invaluable when data availability is low. While this has been demonstrated in certain technology areas, adopting such an approach is new in machine learning (ML) applications in chemistry, except for some pre-training tasks. In drug discovery, predicting binding energy between proteins and ligands is crucial. Many ML-based studies have been proposed to predict protein-ligand binding affinity using existing experimental data. However, these models suffer from inherent biases. Recent efforts have produced PLAS-20k, a synthetic dataset of multiple protein-ligand complex (PLC) conformations generated using molecular dynamics (MD) simulations as a viable option to complement existing experimental data and improve binding affinity prediction. For the binding affinity prediction task, we employ Pafnucy, a deep convolutional neural network, and propose using multiple structures for each PLC from PLAS-20k for training. We compare four different statistical and ML-based result-aggregation techniques. This work demonstrates the utility of dynamic datasets in enhancing binding affinity predictions, laying the foundation for future improvements in predicting similar protein properties using synthetic datasets and more sophisticated models and methods. We propose that physics-based synthetic datasets can significantly help develop more accurate data-driven methods.

**Scientific contribution**

This study shows that incorporating synthetic molecular dynamics data improves deep learning models for protein–ligand binding affinity prediction beyond static experimental structures. By systematically evaluating frame selection and prediction aggregation strategies, we demonstrate that training on diverse conformational snapshots significantly enhances generalization and accuracy. Our results highlight that dynamic synthetic datasets can enable deep learning models to outperform conventional methods such as MM-PBSA while remaining computationally efficient.

## Introduction

Deep learning (DL) has become an indispensable tool that enables learning patterns and deriving information from observations regarding various properties of the studied systems. However, DL models often require immense amounts of well-curated data to accurately grasp the properties they are being trained for and generalize effectively. Unfortunately, real-world data frequently presents challenges such as scarcity, human error, and subjectivity [1]. These issues with real-world datasets can lead to problems in developing and applying machine learning (ML) models. For instance, supervised learning for autonomous vehicles requires diverse datasets, and instance segmentation performance relies on a vast amount of well-annotated data [2, 3]. It has been shown that these biased or low-data scenarios can be aided by using synthetic data. Synthetic or artificial data can be described as data that, instead of being recorded from purely natural observations, has been generated artificially using various algorithms or simulations to mimic a natural setting or help solve specific tasks [4, 5]. Just as synthetic data can aid learning in areas such as computer vision, even in the fields of human analysis and health care, it can help meet the demand for large-scale and publicly available datasets [6, 7]. However, synthetic data is less commonly utilized in fundamental sciences, with most of its usage being in pre-training various models [8–11]. As such, there is plenty of scope to leverage synthetic datasets in domains like molecular science, such as aiding in predicting protein-ligand binding affinity.

Identifying molecules that effectively bind to specific receptors is a pivotal challenge in drug design [12–14]. Protein-ligand interaction scoring functions, crucial for assessing the binding efficacy of various ligands with a given protein receptor binding pocket, directly impact the identification and optimization of potential therapeutic compounds [14–20]. Traditional physics-based approaches like molecular dynamics (MD) simulations [21, 22] and enhanced sampling protocols such as replica-exchange MD (REMD) [23, 24], random accelerated MD (RAMD) [25, 26], umbrella sampling [27, 28], steered MD [29, 30], and metadynamics [31–33] have been widely used to calculate binding affinities of PLCs [34]. Besides these, more generalized computational approaches involve approximate methods like molecular-mechanics Poisson-Boltzmann surface area (MM-PBSA) [14, 35, 36]. However, these methods can be computationally expensive and time-consuming, limiting their scalability for high-throughput screening exercises [37]. Simple scoring functions are typically used to virtually screen large libraries via docking; however, such methods are very approximate [38]. In recent years, ML-based scoring functions have been used in the hope that they will be more accurate and efficient [37, 39].

ML and DL approaches have emerged as preferred tools for developing the scoring functions [37, 40–46], leveraging publicly available protein-ligand binding affinity datasets [47, 48] for accurate and faster predictions. However, despite the advancement of DL models in the drug design domain, it has been observed that the datasets used to train these models themselves harbor latent biases that impact their practical utility, particularly in virtual screening [49–53]. These biases have been systematically investigated, emphasizing the significance of accounting for sequence, protein-ligand interaction, and pocket structure similarities when constructing data splits. The analysis also sheds light on the observed high performance of protein-only and ligand-only models in some datasets, indicating that the models were seemingly not learning the interactions between the ligands and proteins [49]. Another bottleneck in binding affinity prediction is that most datasets feature only the static structure of PLCs [54, 55], while the actual affinities generally depend on protein-ligand thermodynamic ensembles. These challenges show that models developed based on limited experimental data are not robust. However, determining experimental binding affinities at a large scale involving diverse proteins/ligands is expensive, and increasing the size of the dataset is not feasible. Hence, synthetic data approaches could be employed to improve binding-affinity datasets, similar to how they are used in fields like computer vision [1].

MD data can be utilized as a viable source of synthetic data. Dynamic information captured by MD simulations can provide a more comprehensive representation of the binding process, improving prediction accuracy. The graph-based DL model Dynaformer utilized this fact and leveraged geometric features from MD trajectories to outperform static models [40]. Beyond direct supervised use of MD trajectories, the authors of ProtMD have also explored self-supervised pretraining approaches: an equivariant graph-matching network pretrained on MD trajectories via a prompt-based denoising task and a snapshot-ordering task, whose learned representations are subsequently linearly probed or fine-tuned for binding-affinity and ligand-efficacy prediction [56]. Recent studies have published systematically computed datasets such as PLAS-5k and PLAS-20k, comprising binding affinity derived from MM-PBSA of about 5000 and 20,000 PLCs, respectively, with trajectories obtained from MD simulations [55, 57]. These datasets pave the way for utilizing the dynamic information of simulated trajectories for a heterogeneous and larger subset of existing PLCs to enhance the accuracy and generalizability of ML-aided binding affinity predictions. In our work, we utilize the Pafnucy model [58] and train it on about 6000 unique PLCs from the PLAS-20k dataset to examine one aspect of the dataset, which is the utility of the dynamic trajectories (synthetic data) to improve the model performance.

Our study demonstrates how using synthetic data from the MD simulations can improve the performance of ML models compared to static structures. We harness a deep convolutional neural network, Pafnucy, to predict the binding affinities of the PLCs. We conduct extensive experiments to evaluate different methods of selecting snapshots or frames from the available MD trajectories in PLAS-20k, assess the impact of varying the number of frames on prediction accuracy, and compare various aggregation techniques for combining predictions from multiple frames. Our results showcase a significant improvement in model performance upon training it on a larger number of frames per PLC with appropriate frame selection methods. These results suggest that incorporating dynamic data through simple data augmentation can significantly enhance the predictive power of DL models, providing a basis for future research in this area and exploring the possibilities of leveraging synthetic datasets in drug discovery and other ML tasks in fundamental sciences.

## Methods

### Data

#### Dataset

The PLAS-20k dataset is derived from MD simulations and offers an opportunity for integrating MD simulations with ML techniques [57]. With close to 19,500 protein-ligand structures, this dataset encompasses a wide range of PLCs. This information allows us to utilize the dynamic nature of biomolecular systems and incorporate a broader range of data in ML applications. The binding affinities reported in this dataset, computed with the MM-PBSA method and averaged over multiple simulation runs per PLC, are segregated into polar and non-polar components and accompanied by the respective MD trajectories. In addition to the PLAS-20k dataset, we also use the PDBBind dataset [19] to obtain the experimental (ground state) binding affinity of the selected PLCs.

#### Data preparation

The PLAS-20k dataset provides 200 frames (MD trajectory snapshots) generated through five independent MD simulation runs for each PLC. These 200 frames are drawn from the production phase of each MD run, after the system has undergone energy minimization and equilibration [55, 57]. These frames are stored as PDB files containing the spatial coordinates for both protein and ligand structures. Since the Pafnucy model, which is being used for the binding affinity prediction task, also requires protonation states, partial charge information, and the separation of protein and ligand structures into individual files in MOL2 format, preprocessing of the data was required. We supplemented the protonation states and partial charge information by incorporating the PRMTOP parameter files of the PLCs, which also contained the connectivity, atom-type, and residue-type information, along with the corresponding PDB files. As the original PDB files store both protein and ligand structures in a single file, we separated these into distinct files. We utilized the VMD software [59] to achieve both of these tasks using TCL scripts, and for each PLC, we generated 200 MOL2 files for the protein structure and 200 MOL2 files for the ligand structure.

The PLAS-20k dataset reports the binding affinities in the units of kcal/mol. We converted the binding free energies to align with the Pafnucy model’s prediction of binding affinities in terms of $-\log (K_d)$ using the Eq. [60]:

1 $$\begin{aligned} \Delta G_{\text {Binding}} = 2.303RT \cdot \log (K_d) \end{aligned}$$

Here, $K_d$ represents the dissociation constant for each PLC, R is the universal gas constant, and T denotes the absolute temperature in Kelvin.

The Pafnucy model utilizes 3D grids as input, with each grid point characterized by 19 atomic features. These features were computed from the MOL2 files using Open Babel [61] and the tfbio.data package [58]. The feature data and corresponding binding affinity labels were stored in HDF files to train the ML model. Approximately 6000 PLCs were selected for training and testing the model after the preprocessing stage. This subset corresponds to all PLAS-20k PLCs for which an experimental binding affinity was available in PDBBind (Section Dataset), as this is the ground-truth label used for supervised training. A list of the specific PLCs used is provided in our code repository for reproducibility.

### Benchmarks

As described in PLAS-5k and PLAS-20k studies [55, 57], the initial system preparation involves the preparation of the complexes of proteins and ligands. While these complexes are further subjected to energy minimization, we train Pafnucy on these single-frame PLCs obtained before the energy minimization stage (essentially experimental structures) for the baseline model. This baseline could allow us to evaluate the effectiveness of the availability of multiple frames in improving the data quality.

In addition to the baseline DL model, we also utilize the MM-PBSA values (obtained by harnessing the Poisson-Boltzmann equation for depicting free energy differences between two states of a system [35]), available as part of the PLAS-20k dataset, for comparing the performance of our multi-frame approach.

### Trajectory frames

As mentioned, the PLAS-20k dataset consists of 200 frames or snapshots obtained from the MD simulations for each PLC. While all of these frames can be used to train the Pafnucy model, training the model on all 200 frames is computationally expensive and time-consuming. Also, many snapshots may be closer in overall structure to each other. Therefore, we selected a smaller sample set from these 200 frames per PLC. In this study, we compare the effects of the number of frames chosen to train the model and the method used to choose these frames from all the available ones. Both the number of frames and the method of selection influence the diversity of the conformations captured by the model. We look at the different techniques of frame selection below.

#### Randomized selection

In this method, we randomly select a specified number of frames from the total number of frames for each PLC. This way of randomly selecting a subset of frames does not impose any specific criteria for the selection. However, it provides no control over whether the chosen frames are similar to one another.

#### Starting frames selection

This method allows us to evaluate how a continuous selection of frames performs compared to more distributed frame selections by choosing the required quantity of frames from only the initial trajectory run corresponding to each PLC. However, it also suffers from not considering the possible diversity in the remaining frames.

#### Uniform selection

In this approach, we select frames at uniform intervals across the 200 frames in order to capture a more representative set of frames. This could potentially help capture the overall distribution of conformations rather than focusing on one particular region of the trajectory. After determining the interval size, we select frames spaced by this interval, ensuring that the total number of selected frames does not exceed the predefined number per PLC.

#### Clustered selection

This method uses clustering algorithms to group similar frames and choose frames from these clusters. For each PLC separately, we first compute the pairwise root mean square deviation (RMSD) of the coordinates across the 200 frames. Then, we use the K-medoids algorithm [62] on the resulting 200x200 matrix of pairwise RMSD values to cluster the frames. For our purposes, we find K-medoids to be more suitable than methods such as K-means [63], as the K-medoids algorithm selects actual data points as cluster centroids and is more robust to outliers [64]. Since the number of clusters is not predetermined, we use the silhouette-score metric to determine each PLC’s optimal number of clusters [65, 66]. Once the clusters are formed, frames are arbitrarily chosen from within each cluster. The number of frames selected from each cluster is in proportion to the size of the cluster and the total number of frames decided to be chosen.

### Model

#### Pafnucy

Pafnucy is a deep convolutional neural network (CNN) model consisting of 3-dimensional convolutional layers and fully connected dense layers, which is used to estimate the binding affinity of ligand-receptor complexes [58]. It represents each PLC as a 4D tensor containing the x, y, and z spatial coordinates and a vector of 19 features, such as atom type, hybridization, partial charge, etc. The input molecule first passes through three 3D convolutional layers, with 64, 128, and 256 filters combined with max-pooling layers. The output is then flattened and passed through three fully connected dense layers containing 1000, 500, and 200 neurons, respectively. A dropout rate of 0.5 is applied to all dense layers to prevent over-fitting. When tested on the PDBbind database v. 2016 core set [19], Pafnucy obtained a root mean squared error (RMSE) of 1.42 and a Pearson correlation of 0.78. The model also outperformed all 20 scoring functions provided by the CASF-2013 ‘scoring power’ benchmark [67].

**Fig. 1:**
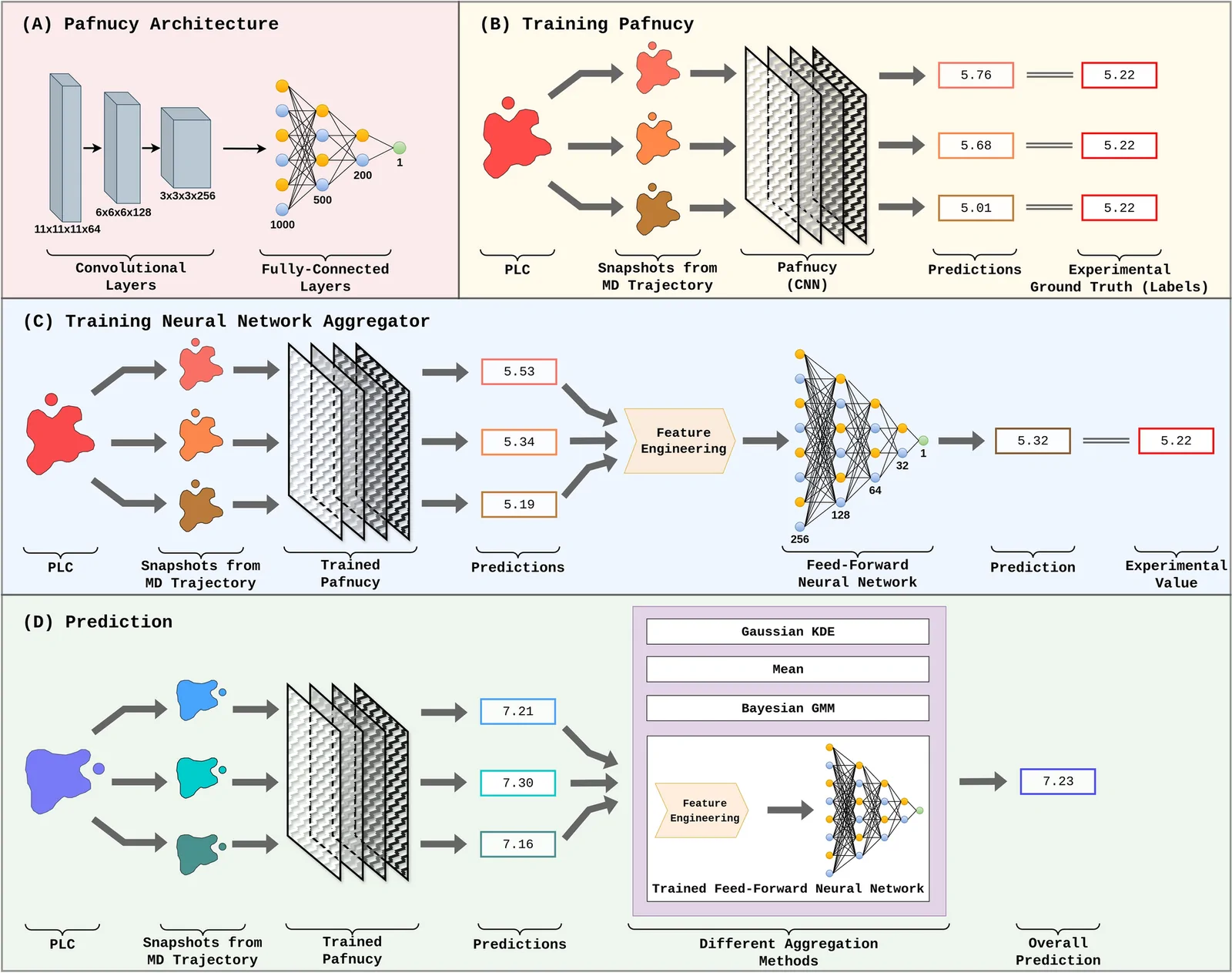
Schematic diagram of the modified binding affinity prediction pipeline. **A** The Pafnucy architecture serves as the primary deep convolutional neural network for predicting binding affinity. **B** During training, multiple frames from each PLC are provided as separate inputs to the model, which is trained to predict the same experimental binding affinity for all frames. **C** Subsequently, a feed-forward neural network is trained to aggregate the CNN outputs corresponding to each PLC. **D** Once fully trained, the complete model can take multiple snapshots from a single PLC and, using a specified aggregation method, combine their predictions to produce a final binding affinity estimate

#### Modified pipeline

We utilized the Pafnucy model in our study to predict binding affinity values by feeding multiple frames of a PLC into the model instead of using a single frame. One can refer to Table 3 in Appendix B for the hyperparameters. We performed a 9:1 train-test split of the dataset. We ensured that there is no PLC overlap between the train and test sets, and that all frames belonging to a single PLC are assigned entirely to either the training or test set. This split is therefore performed at the protein–ligand complex (PLC) level. We note that a complex-level split does not by itself guarantee protein-level separation. Because the PLCs are drawn from PDBBind, which contains many complexes of the same protein bound to different ligands, a given protein sequence may appear (with different ligands) in both splits. Quantifying this, the mean of the per-protein maximum sequence identities between a test protein and the training set is 92.7%, and 77.5% of test proteins have an identical-sequence counterpart in the training set (full analysis in Appendix D). As shown in Appendix D (Fig. 4), the improvement from multi-frame augmentation over the single-frame baseline is nonetheless preserved within every sequence-identity stratum, including test proteins with low identity (< 30%) to the training set. Therefore, the qualitative conclusions of this study are not an artifact of protein redundancy, although the absolute error values should be interpreted in light of it. For ligands, we observed a Jaccard similarity of only 0.035 between the training and testing sets. Each PLC was selected based on the number of frames and the selection method (Section Trajectory frames), allowing for performance comparison across these different approaches.

As shown in Fig. 1, basic data augmentation was applied for training by assigning the same binding affinity value corresponding to the reported experimental ground-truth to every frame of a given PLC. These frames were treated as independent molecular complexes when input into the architecture. This architecture then maps each frame individually to the specified binding affinity. For testing, we input the various frames of the complexes into the trained model. The model outputs a predicted binding affinity for each frame in terms of $-\log (K_d)$. We then convert these values back to kcal/mol (Eq. (1), Section Data preparation). Since multiple frames were provided for each protein-ligand complex, we obtained several predicted values for each complex. To generate a single final prediction, we aggregated the results using methods such as Gaussian kernel density estimation, arithmetic mean, Bayesian Gaussian mixture models, and feed-forward neural networks (Section Prediction aggregation). Following this, we evaluated model performance by calculating the mean squared error (MSE) and Pearson correlation coefficient ($r_p$) to assess accuracy.

#### Cross-architecture validation

Pafnucy (Section Pafnucy) was selected for this study as a simple, well-characterized architecture that lets us isolate the effect of the data pipeline (frame selection, frame count, aggregation) from architectural complexity, rather than for state-of-the-art performance. Several more recent architectures for this task are discussed in Section Introduction. To test whether the trends reported in Section Results and discussion generalize beyond this specific architecture, we additionally trained and evaluated the Geometric Interaction Graph Neural Network (GIGN) model [68] using the same frame-selection and aggregation protocol on the same dataset. GIGN is a more recent graph neural network that explicitly encodes covalent/non-covalent interactions and is rotation/translation-invariant by construction. Full methodological details and hyperparameters (Table 4) are presented in Appendix C. The purpose of this cross-architecture experiment is to test whether the qualitative trends associated with frame selection, frame count, and aggregation transfer to a structurally different model, not to maximize absolute predictive accuracy or to rank the two architectures against each other. As detailed in Appendix C, the two are trained under different protocols and their absolute error values are not directly comparable.

### Prediction aggregation

When multiple frames of the same protein-ligand complex are provided to the model, a predicted binding affinity for each frame is produced separately. As a result, multiple binding affinity predictions are made for the same complex. Therefore, we aggregated the predictions for each complex to produce a single output and evaluated the performance of three different aggregation methods, as discussed below.

**Fig. 2:**
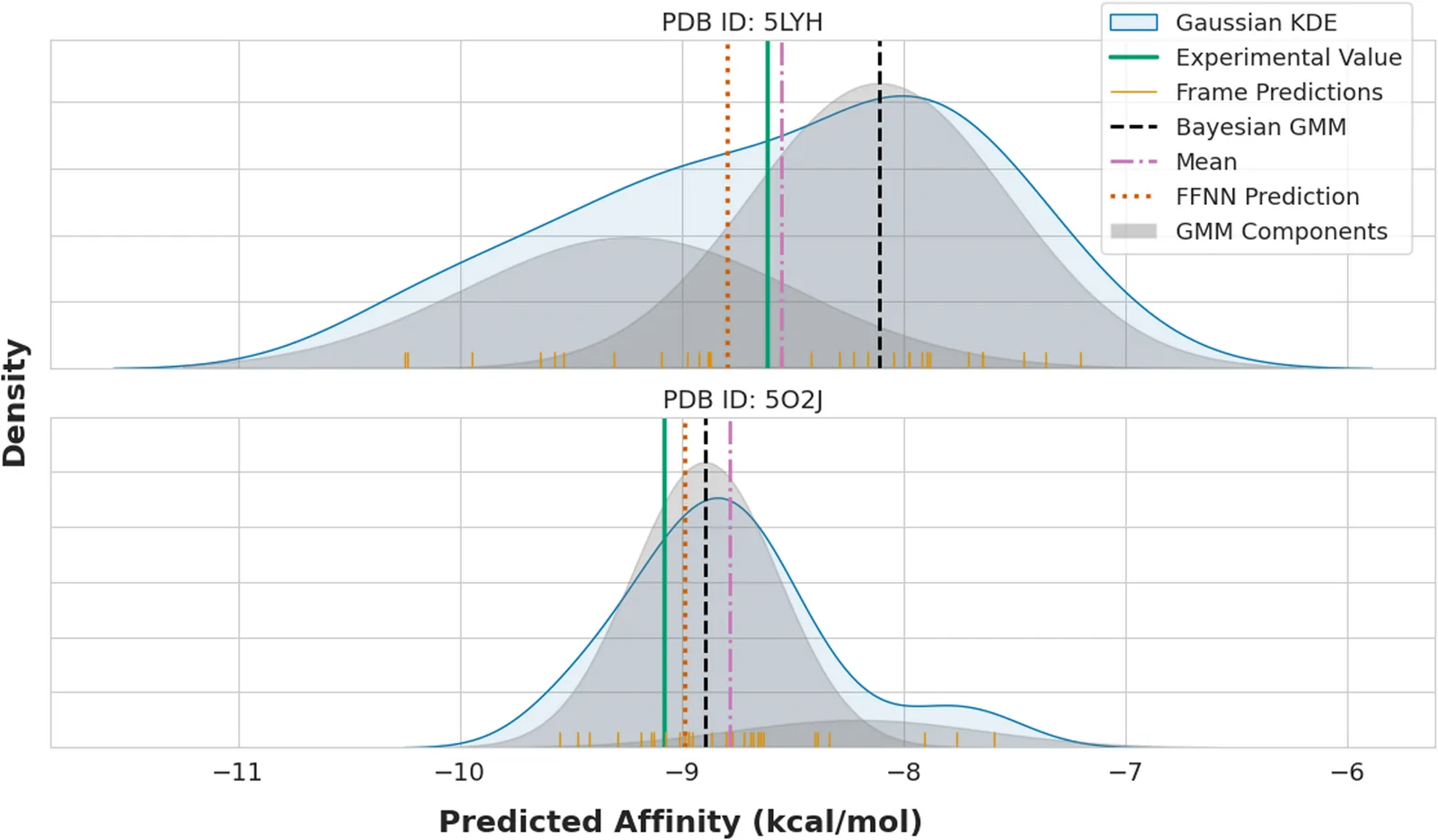
Visualization of different aggregation techniques for model predictions. From the outputs of a model trained on 30 frames per PLC, we display the predictions made for two randomly selected PLCs (5LYH and 5O2J) from the data. Each PLC has 30 different outputs for the respective frames. The mean prediction (pink dashed line) is made by simply taking the arithmetic mean of these values. On the other hand, the final prediction made by Gaussian KDE is the binding affinity value, where the KDE curve (blue curve) has the highest peak. For the Bayesian GMM approach, various Gaussian components are identified (as shown by the gray-shaded regions). The most significant one is selected, and its mean (black dotted line) is used to make the final prediction. Predicted $\Delta G_{\text {Binding}}$ from our trained FFNN (orange dotted line) has also been compared

#### Gaussian kernel density estimate

Kernel Density Estimation (KDE) calculates the probability density function of a random variable by assigning a kernel to each data point. A kernel is a real-valued, non-negative function where the area under the curve equals one, and it is usually symmetric around the data point to which it is assigned. The estimated overall density is obtained by first summing the values of the kernels across the entire range of data and then normalizing the sum by the number of data points [69, 70].

In Gaussian KDE, each kernel is modeled as a Gaussian function centered at the corresponding data point. A tunable parameter called the bandwidth controls the variance of these kernels.

2 $$\begin{aligned} K(x) = \frac{1}{n h} \sum _{i=1}^{n} \mathcal {K} \left( \frac{x - x_i}{h} \right) \end{aligned}$$

Here, K(x) represents the overall KDE at point x, $\mathcal {K}$ is the Gaussian kernel, n is the number of data points, and h is the bandwidth.

In this study, we take the predicted binding affinities for each PLC and compute the Gaussian KDE for these predictions, as shown in Fig. 2. Then, we select the binding affinity corresponding to the peak of this density estimate as the final binding affinity for each complex.

#### Mean

Calculating the arithmetic mean of the data points is a commonly used aggregation method. Using this standard method of central tendency, we aggregate the predicted binding affinities for each PLC by evaluating their unweighted arithmetic mean (Fig. 2).

3 $$\begin{aligned} \text {Mean} = \frac{1}{n} \sum _{i=1}^{n} x_i \end{aligned}$$

Here $x_i$ represents the predicted binding affinity for the i-th selected frame, and n is the number of frames chosen for a given complex.

#### Bayesian gaussian mixture model

Gaussian Mixture Model (GMM) assumes that all the data points are generated from a mixture of a finite number of Gaussian distributions to cluster points. Based on this assumption, it assigns a probability of belonging to a particular cluster to each data point, thereby acting as a soft clustering algorithm. This approach contrasts with hard clustering methods like K-means, which assign each data point to exactly one cluster. The GMM algorithm utilizes the Expectation-Maximization (EM) algorithm to estimate parameters such as mean, covariance, and weight of each component [71, 72].

In many cases, the number of clusters to be formed is unknown, which can be a challenge. This issue can be tackled possibly by using a Bayesian GMM, which applies variational inference to calculate the posterior distributions of the parameters of the Gaussian mixture [73, 74].

In our work, we fit a Bayesian GMM on the output predictions that identifies different Gaussian components to obtain predicted binding affinities for each PLC. Once these components are identified, the one with the highest weight is selected, as shown in Fig. 2, and the mean of this component is taken as the final aggregated binding affinity for the respective complex.

#### Feed-forward neural network

A Feed-Forward Neural Network (FFNN) is a type of artificial neural network where data travels in a single direction, from the input to the output, without forming any loops. It’s composed of an input layer, one or more intermediate hidden layers, and an output layer. Each neuron in these layers applies a non-linear function to the weighted sum of its inputs.

Our work employs an FFNN with four hidden layers of sizes 256, 128, 64, and 32. Each layer uses the SiLU (Sigmoid Linear Unit) activation function, and dropout regularization is applied at each layer with rates of 0.4, 0.3, 0.2, and 0.1, respectively. The network is trained on the frame-level binding affinity predictions generated by Pafnucy for the training set. We also include statistical features such as the minimum, maximum, mean, median, and standard deviation of the frame-level predictions to enhance representation. All input features are scaled using a MinMax scaler. This FFNN learns to map the set of frame-level predictions for each PLC to a single aggregated binding affinity, providing a machine learning-based alternative to rule-based aggregation methods.

## Results and discussion

**Fig. 3:**
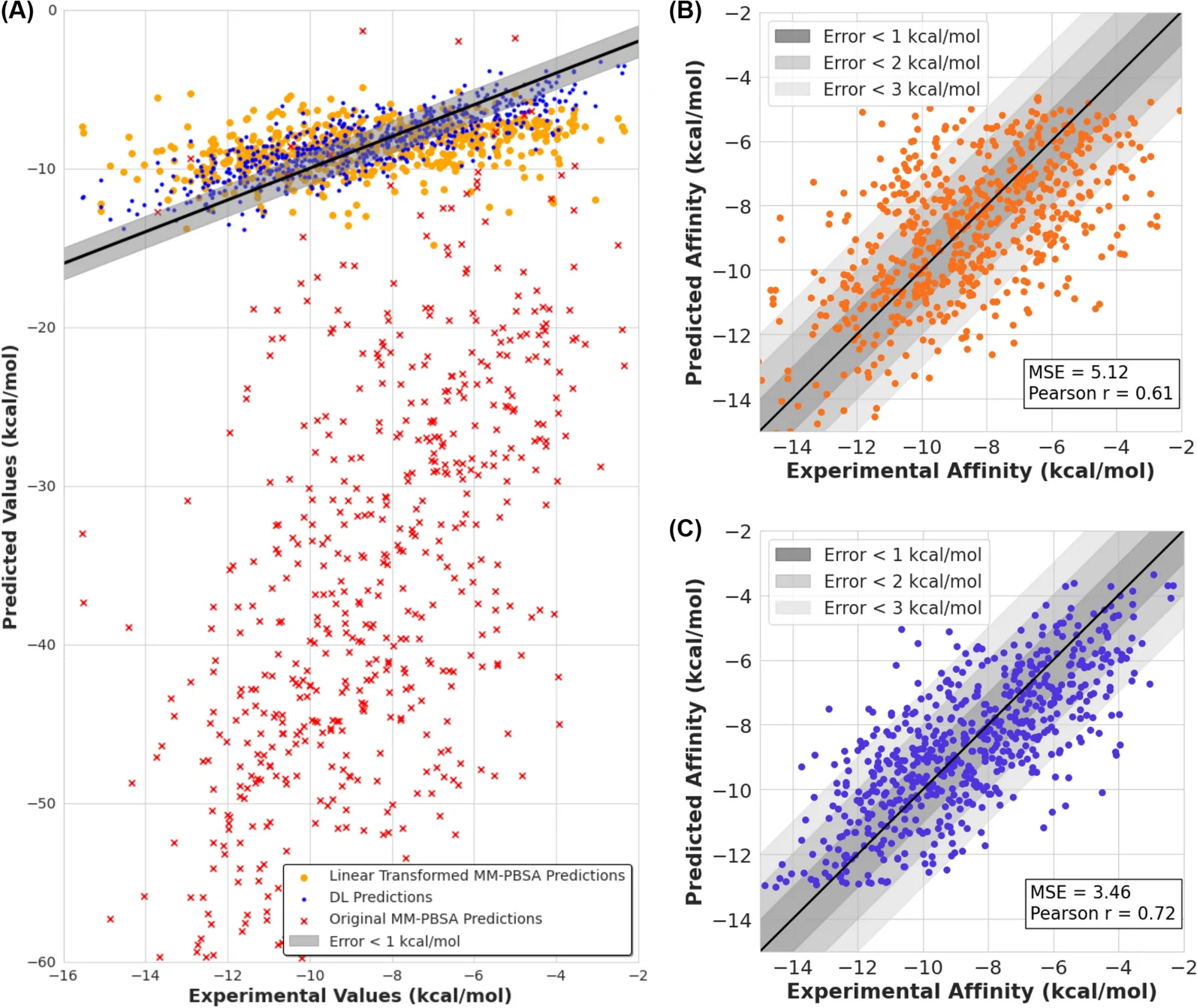
Scatter plots for binding affinity predictions made by different approaches. **A** Comparison between the accuracy of the multiple frame DL approach, the original naive MM-PBSA, and the linearly transformed MM-PBSA approach. The linear transformation improves the accuracy compared to the original MM-PBSA, although the DL approach still outperforms both. **B**, **C** Predictions made respectively by the baseline model (**B**) (section Benchmarks), and the model trained using 30 frames per PLC selected using the clustering approach (section Clustered selection) and aggregated using the feed-forward neural network (**C**). The baseline predictions are dispersed away from the ideal values more than those of the multiple frames approach

### Comparison with MM-PBSA and baseline

The performance of the traditional MM-PBSA technique to calculate the binding free energies of the various PLCs is much worse than that of the deep learning model trained using multiple frames. It is well known that naive MM-PBSA free-energy estimates carry a large systematic offset relative to experimental values [35, 75]. The uncorrected predictions in our dataset have a test-set MSE of 1176.0  $(\text {kcal} \cdot \text {mol}^{-1})^2$ (Table 1, footnote), reflecting this offset. To enable a fair comparison, we apply a standard linear transformation (slope/intercept fit on the training set; full derivation in Appendix A) to the MM-PBSA predictions before comparing them to the DL approach. As shown in Fig. 3A, even after this correction, the linearly-transformed MM-PBSA predictions show a larger deviation from the experimental values, and a higher test-set MSE (5.38  $(\text {kcal} \cdot \text {mol}^{-1})^2$), than the multi-frame DL approach (best test MSE 3.46).

When comparing the method of the baseline approach, which uses complexes prior to energy minimization (experimental structures) from the PLAS-20k dataset without multiple frames, with those that utilize several frames and aggregation techniques, we see that the former’s predictions are more scattered away from the ideal predictions than the latter’s (Fig. 3B, C). The baseline shows higher MSE (5.1  $(\text {kcal} \cdot \text {mol}^{-1})^2$ for the testing set) and lower $r_p$ (0.6 for the testing set) values, showcasing the advantage of using multiple frames for more accurate binding affinity predictions (Tables 1 and 2).

### Effectiveness of frame selection strategies

Out of the four methods we used for selecting frames for each PLC, we observe that the approaches that rely on frames that are either randomly selected or selected from the initial trajectory run have low MSE and high $r_p$ for the training set. However, they perform notably poorer on the testing set (Tables 1 and 2). Choosing frames specifically from the initial trajectory coordinates likely suffered due to these being too similar, leading to an over-representation of a limited conformational region. Similarly, it seems that the random selection also resulted in insufficient representation of the conformational diversity for the chosen PLCs. These results suggest that learning representation of similar frames is more straightforward for Pafnucy, improving training performance but reducing generalizability to unseen testing data.

Based on the results in Tables 1 and 2, we believe the uniform selection method improves upon the previous two frame selection approaches by sampling evenly across the entire trajectory, possibly capturing a broader range of conformations. However, we observe that the best performance is achieved using the clustered selection method. Unlike the previous approaches, this method specifically groups similar frames together and selects representatives from these groups, possibly giving a higher probability of having a more structurally diverse set of input. Therefore, the clustering approach supports the testing data’s lowest MSE (3.5  $(\text {kcal} \cdot \text {mol}^{-1})^2$) and highest $r_p$ (0.7) values.

**Table 1:** Pafnucy mean squared errors (in units of  $(\text {kcal} \cdot \text {mol}^{-1})^2$) obtained from the analyses protocols (see Sections Benchmarks and Trajectory frames), evaluated on the training and testing datasets

Approach	Number of frames	Train set MSE					Test set MSE				
		Direct	KDE	Mean	GMM	ANN	Direct	KDE	Mean	GMM	ANN
MM-PBSA$^{1}$		5.49					5.38				
Baseline		3.04					**5**.**12**				
Random	1	4.59					5.91				
	10		1.23	1.14	1.14	1.19		5.11	4.83	4.84	4.99
	20		1.30	1.28	1.29	1.20		4.46	4.37	4.41	4.53
	30		1.44	1.43	1.43	1.40		4.43	4.30	4.32	4.42
Starting	1	5.13					**5.01**				
	10		1.17	1.15	1.15	0.96		4.54	4.54	4.55	4.53
	20		1.36	1.36	1.35	1.18		4.32	4.30	4.29	4.40
	30		1.84	1.86	1.85	1.49		4.67	4.64	4.64	4.77
Uniform	10		1.55	1.52	1.52	1.19		4.81	4.61	4.63	4.67
	20		1.49	1.48	1.48	1.22		4.78	4.61	4.65	4.81
	30		1.41	1.42	1.41	1.25		4.00	3.88	3.96	3.97
	40		1.63	1.64	1.64	1.56		4.55	4.43	4.50	4.58
Clustered	10		1.25	1.18	1.18	1.12		4.44	4.17	4.20	4.22
	20		1.27	1.27	1.27	1.16		4.01	3.81	3.87	3.90
	30		1.50	1.49	1.48	1.40		**3.63**	**3.50**	**3.54**	**3.46**
	40		1.77	1.77	1.77	1.62		4.09	3.99	4.09	4.10
	50		1.76	1.77	1.77	1.58		**3**.**66**	**3**.**67**	**3**.**71**	**3**.**81**
	60		1.73	1.75	1.75	1.50		3.93	3.86	3.88	4.15

$^{1}$The MM-PBSA MSE values are reported for the linearly transformed approach as described in Appendix A. (MSE corresponding to the original MM-PBSA values for the test set is 1176.0  $(\text {kcal} \cdot \text {mol}^{-1})^2$)

In each test-set column, the second-best-performing method is shown in bold, and the best is both bold and underlined

### Effect of frame quantity on performance

On varying the number of frames selected per PLC for different deep learning models and comparing their performances, we observed that, similar to using a static structure, using a single frame results in higher MSE across the randomized selection, starting-frames selection, and baseline approaches than when multiple frames are used (Table 1). While increasing the number of frames generally leads to higher training error up to 40 frames, training error plateaus thereafter (e.g., 1.77, 1.77, and 1.75  $(\text {kcal} \cdot \text {mol}^{-1})^2$ for 40, 50, and 60 frames, respectively, using mean aggregation, clustered selection), while test error does not improve further and, in most configurations, is worse than at 30 frames. We extended our experiments up to 60 frames using the clustered selection approach (our best-performing strategy) to verify this trend. Rather than a continued decline, performance beyond 30 frames fluctuates (test MSE of 3.99, 3.67, and 3.86  $(\text {kcal} \cdot \text {mol}^{-1})^2$ at 40, 50, and 60 frames, respectively, versus 3.50 at 30 frames), but in no case surpasses the 30-frame result within the range tested. In our experiments, we therefore obtain the best performance (i.e., lowest MSE and highest $r_p$) on unseen test data by selecting 30 frames using the clustered selection approach. Even for the uniform selection approach, we notice that selecting 30 frames leads to better results.

A plausible explanation for this performance trend of the different models is that with limited data, the models tend to overfit and result in low training error but high test error. Overfitting is reduced as the number of frames increases up to a point, and the model begins to generalize better with lower test errors [76, 77]. However, this benefit does not continue indefinitely: beyond 30 frames, test performance does not improve further and instead fluctuates without exceeding the 30-frame optimum. This is possibly due to the introduction of redundant or highly similar frames at higher counts, which add training cost without adding genuinely new conformational information, or due to a mismatch between the increased input diversity and the fixed capacity of the CNN/aggregation pipeline [78]. To elaborate, Pafnucy projects each complex onto a fixed 1Å grid (Table 3), meaning that conformational differences smaller than the grid spacing are largely flattened out, causing closely related frames to map to near-identical input tensors. Moreover, because Pafnucy is not rotation-invariant, every input is presented in 24 rotations during training (Table 3). A 30-frame training set thus already yields 720 distinct input tensors per complex, capturing much of the diversity that additional frames would otherwise introduce. As such, Pafnucy showcases a capacity limitation rather than a data shortage, and its training error plateaus beyond roughly 40 frames instead of continuing to decline. Consequently, the optimal frame count depends heavily on both the network architecture and its input representation, a conclusion supported by the higher optimum observed for the rotation-invariant, graph-based GIGN model (Appendix C). Hence, while increasing the number of frames improves accuracy up to an architecture- and selection-strategy-dependent point, identifying the optimal number of frames is likely necessary to achieve the best performance [79].

**Table 2:** Pafnucy Pearson correlation coefficients ($r_p$) of different methods evaluated on the training and testing datasets

Approach	Number of Frames	Train Set Pearson $r_p$						Test Set Pearson $r_p$				
		Direct	KDE	Mean	GMM	ANN		Direct	KDE	Mean	GMM	ANN
MM-PBSA		0.54						0.49				
Baseline		0.78						**0.61**				
Random	1	0.64						0.54				
	10		0.92	0.92	0.92	0.92			0.63	0.65	0.65	0.64
	20		0.92	0.92	0.92	0.92			0.67	0.68	0.68	0.67
	30		0.91	0.91	0.91	0.91			0.66	0.67	0.67	0.66
Starting	1	0.58						**0**.**55**				
	10		0.93	0.94	0.94	0.94			0.62	0.62	0.62	0.62
	20		0.93	0.93	0.93	0.92			0.66	0.66	0.66	0.66
	30		0.90	0.90	0.90	0.90			0.65	0.65	0.65	0.65
Uniform	10		0.91	0.92	0.92	0.92			0.63	0.65	0.65	0.64
	20		0.91	0.92	0.91	0.92			0.64	0.66	0.66	0.65
	30		0.91	0.92	0.91	0.92			**0**.**70**	0.70	**0**.**70**	0.70
	40		0.89	0.90	0.90	0.90			0.64	0.65	0.65	0.65
Clustered	10		0.92	0.92	0.92	0.92			0.65	0.67	0.67	0.67
	20		0.92	0.92	0.92	0.92			0.69	**0**.**71**	**0**.**70**	**0**.**71**
	30		0.91	0.91	0.91	0.91			**0.71**	**0.72**	**0.72**	**0.72**
	40		0.88	0.89	0.88	0.89			**0**.**70**	**0**.**71**	**0**.**70**	0.70
	50		0.89	0.89	0.89	0.89			**0**.**70**	0.70	**0**.**70**	0.70
	60		0.90	0.90	0.89	0.90			0.67	0.68	0.68	0.68

In each test-set column, the second-best-performing method is shown in bold, and the best is both bold and underlined

### Influence of aggregation techniques on prediction accuracy

We compared the performance of four methods for aggregating the predictions from multiple frames: Gaussian KDE, arithmetic mean, Bayesian GMM, and a Feed-Forward Neural Network (FFNN). Based on the testing data MSE values, the simple mean method consistently outperforms the KDE and Bayesian GMM approaches in most cases. For example, under the clustering-based frame selection approach for 30 frames, the mean method yields an MSE of 3.50, compared to 3.63 and 3.54 $(\text {kcal} \cdot \text {mol}^{-1})^2$ for Gaussian KDE and Bayesian GMM, respectively (Table 1).

The KDE method, which estimates a probability density function over the predictions and selects the peak value, generally performs worse than the mean and Bayesian GMM methods across most configurations. For the training data, both the mean and Bayesian GMM methods perform comparably—MSEs of 1.52 $(\text {kcal} \cdot \text {mol}^{-1})^2$ each, with the same $r_p$ values of 0.92, under the 10-frame uniform selection strategy. However, on the testing set, Bayesian GMM slightly underperforms compared to the mean (e.g., MSEs of 4.61, 4.63, and 4.81 for mean, GMM, and KDE, respectively), reinforcing the practical advantages of the mean method due to both its performance and computational simplicity.

When introducing the FFNN-based aggregation, we observe that it initially lags behind the statistical methods in most configurations. However, its performance improves significantly as the number of frames increases, especially under the clustering-based frame selection approach. Notably, the FFNN achieves the best overall results—an MSE of 3.46 and an $r_p$ of 0.72—under the clustered 30-frame setup (Tables 1 and 2). Although the FFNN shows lower performance in other frame quantity and selection combinations, it consistently follows the same trend as the statistical methods: error generally decreases with more frames until a certain point, after which it starts increasing. This behavior suggests that the FFNN’s success heavily depends on the quality of initial frame-level predictions generated by Pafnucy. Although the arithmetic mean is still the most practical and reliable aggregation method, the FFNN shows potential under optimized conditions, such as a carefully chosen frame selection strategy and frame count. Importantly, both statistical and machine learning-based aggregation methods highlight similar performance trends, underlining the critical role of selecting the right number of frames and an appropriate frame selection strategy for effective prediction aggregation.

Our results demonstrate that utilizing multiple snapshots of each PLC from MD simulations in a DL model, such as Pafnucy, improves the predictive accuracy compared to solely relying on static structures. Different variations of the Pafnucy model, achieved by training with diverse frame quantities and approaches, can replace methods such as MM-PBSA due to improved results and lower computational costs. Moreover, with sufficient frames and appropriate frame selection and output aggregation techniques, it is seen that the dynamic information in the PLAS-20k could possibly be used more effectively, leading to greater generalizability of the model and higher precision of binding affinity predictions of PLCs. Notably, these trends are not specific to the Pafnucy architecture. Repeating the same frame-selection and aggregation experiments with GIGN, a structurally distinct and more recent graph neural network, yields the same qualitative improvement over a single-frame baseline. The clustered, multi-frame training and simple mean aggregation again outperform the alternatives (Tables 5 and 6, Appendix C). This cross-architecture consistency reinforces that the benefit we observe stems from the synthetic MD data itself, rather than from properties specific to the Pafnucy model. These observations emphasize how crucial synthetic datasets are, particularly those that incorporate dynamic information, in understanding the complexities of the protein-ligand binding process. More generally, this illustrates the potential of synthetic data to advance numerous scientific fields.

## Conclusion

For ML techniques applied in the domains of chemistry and biology, obtaining large amounts of high-quality experimental data for model training is often infeasible. Augmenting the data with synthetic datasets can help in solving such low-data issues. As a test of the usage of synthetic data, this work focuses on the task of protein-ligand binding affinity prediction. Since predicting binding affinity between proteins and ligands is a crucial aspect of drug discovery, several non-ML and ML techniques have been developed to achieve this task. Various ML methods have already shown great promise in improving the efficiency and accuracy of these predictions. However, further enhancement of these models may require moving beyond the traditional usage of static structures usually available in most commonly used datasets. This limitation of static structures could lead to moving on to synthetic datasets with dynamic (MD) trajectories, such as the PLAS-20k dataset. Our findings clearly demonstrate the effectiveness of synthetic data in enhancing data augmentation techniques, significantly improving binding affinity predictions. A cross-architecture check using a second, more recent model (Appendix C) supports the generality of this conclusion beyond the specific architecture used in our main analysis, while also showing that some configuration choices (e.g., the precise optimal frame count) are architecture-dependent and should not be over-generalized from a single model. Overall, our results highlight the invaluable role synthetic datasets can play in future research and applications.

While this study shows the benefits of utilizing the dynamic information present in datasets such as the PLAS-20k by training the simple Pafnucy model, further improvements to the deep learning approach can include making use of more sophisticated prediction models, which could incorporate techniques that allow them to learn the relationships between snapshots of the same PLC. One could also train the models on negative samples, allowing the models to understand the unstable configurations in addition to the stable ones. These improvements could potentially allow DL to generalize even better to unseen PLCs and lead to higher efficiency of the drug discovery pipeline. Besides binding affinity, one could take up similar ideas of utilizing synthetic datasets to study other protein properties and use a combination of experimental and synthetic data to develop better models.

## Figures and Tables

**Fig. 4:**
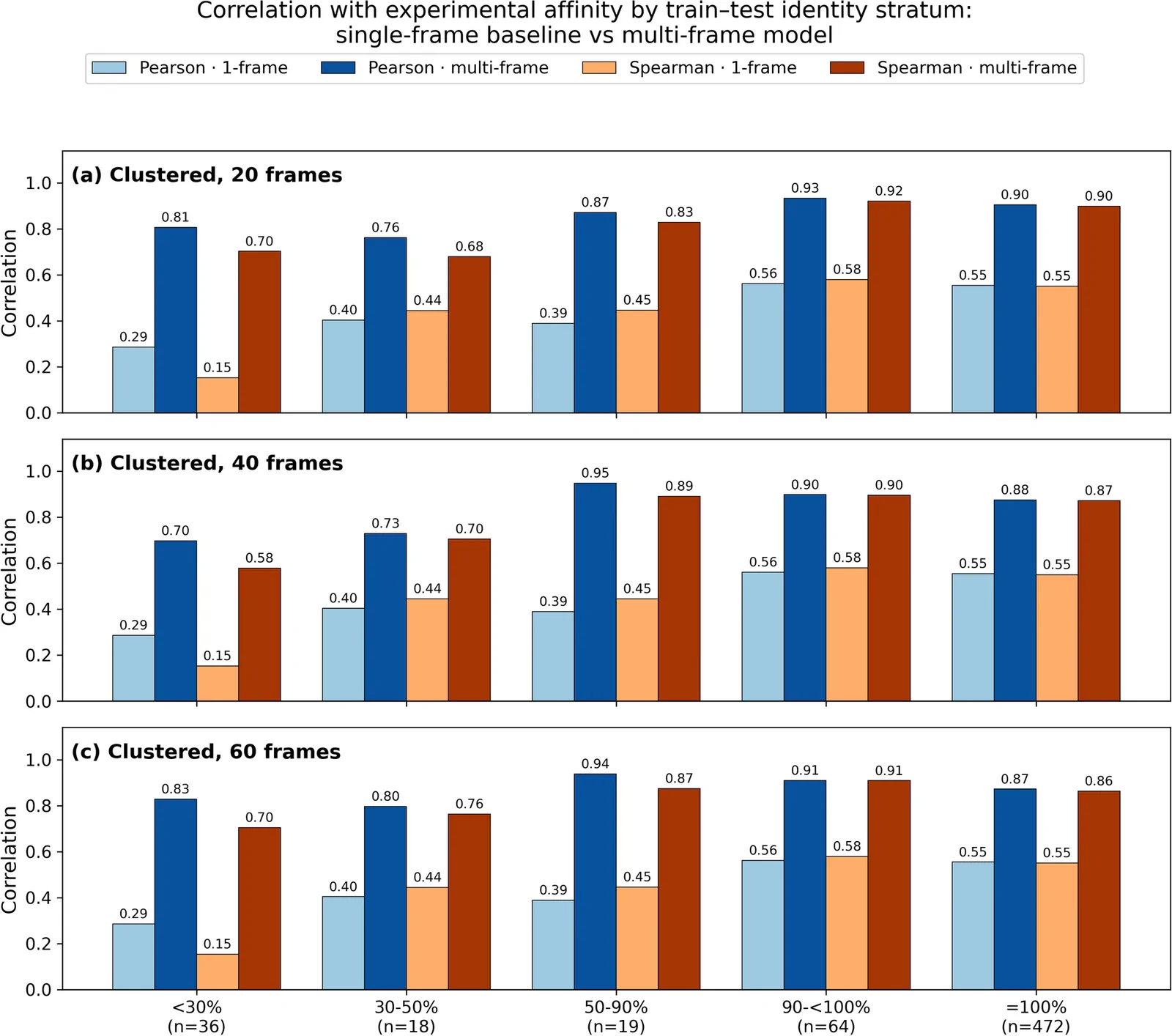
Correlation between predicted and experimental binding affinity, by train-test sequence-identity stratum, for the single-frame baseline and the multi-frame clustered model at (**a**) 20, (**b**) 40 and (**c**) 60 frames. Light bars: baseline (1 frame); dark bars: multi-frame. Blue: Pearson $r_p$; orange: Spearman ρ. The number of test proteins (*n*) in each stratum is shown on the axis. In every stratum and at every frame count, both correlations improve under multi-frame training, including for the low-identity (novel) proteins

## Data Availability

The PLAS-20k dataset used for the analysis is publicly available at https://healthcare.iiit.ac.in/d4/plas20k/about/about.html.

## Appendix A: MM-PBSA linear transformation

Since the predictions made by the naive application of MM-PBSA produced a high deviation from the experimental predictions, as shown in Fig. 3A, we applied a linear transformation to adjust the MM-PBSA output. First, using the training data, we determine the best-fit line of degree 1 for the plot of MM-PBSA predictions versus experimental values. Then, during the inference stage, we utilize the slope and intercept obtained from this best-fit line to linearly transform MM-PBSA output for the testing data, and these transformed values are used as the final predictions.

Let the MM-PBSA predictions from the training data be represented as $\hat{y}_{\text {train}}$, and the corresponding experimental values as $y_{\text {train}}$. The best-fit line for the training data can be expressed as:

$$y_{\text {train}} = m \hat{y}_{\text {train}} + c$$

where m is the slope and c is the intercept. These parameters are obtained by minimizing the least squares error:

$$m, c = \arg \min _{m, c} \sum _{i=1}^{n} \left( y_{\text {train}, i} - (m \hat{y}_{\text {train}, i} + c) \right) ^2$$

Once the slope m and intercept c are determined from the training data, the linear transformation is applied to the MM-PBSA predictions for the testing data $\hat{y}_{\text {test}}$ as follows:

$$\hat{y}_{\text {test}}' = m \hat{y}_{\text {test}} + c$$

where $\hat{y}_{\text {test}}'$ represents the transformed MM-PBSA predictions for the testing data, which are used as the final predictions.

While applying the linear transformation to the original MM-PBSA output changes the mean squared error, it does not affect the Pearson correlation coefficient, which remains the same for both approaches.

## Appendix B: Model hyperparameters

The majority of hyperparameters used for our work are the same as those used by the original Pafnucy model. Table 3 provides a list of hyperparameters along with their values and descriptions. Additionally, we set the random seed to 42 for all tasks involving randomness.

**Table 3:** Pafnucy model hyperparameters, their values, and descriptions

Hyperparameter	Value	Description
grid_spacing	1.0	Distance between grid points
max_dist	10.0	Max distance from complex center
conv_patch	5	Patch size for convolutional layers
pool_patch	2	Patch size for pooling layers
conv_channels	64, 128, 256	Number of filters in convolutional layers
dense_sizes	1000, 500, 200	Number of neurons in dense layers
keep_prob	0.5	Keep probability for dropout
l2	0.001	Weight decay (L2 regularization)
rotations	24	Number of rotations to perform on input
learning_rate	1e-5	Learning rate for the optimizer
batch_size	32	Batch size for training
num_epochs	20	Number of epochs for training

All settings were held fixed across every frame-selection strategy and frame count tested

## Appendix C: GIGN cross-architecture validation

To assess whether the trends reported in Section Results and discussion are specific to the Pafnucy architecture, we repeated the central frame-selection and frame-count experiments using a second, more recent architecture, GIGN [68], on the same dataset, the same frame selections, and the same train/test split used throughout the main text. Throughout this appendix, the GIGN results should be read as a qualitative check on the trends reported in the main text rather than as a head-to-head performance comparison with Pafnucy. The two models were trained under different protocols, and GIGN was not optimized for absolute accuracy on this dataset.

### C.1: Model

GIGN (Geometric Interaction Graph Neural Network) represents a PLC as a graph in which covalent (intramolecular) and non-covalent (intermolecular) interactions are passed through a dedicated heterogeneous interaction layer, rather than being treated identically. The model is invariant to translation and rotation of the input structure by construction, so, unlike Pafnucy, which requires explicit rotational data augmentation (Appendix B), no rotation augmentation is needed for GIGN.

### C.2: Experimental setup

We trained GIGN using the same Baseline, Uniform, and Clustered frame-selection configurations evaluated for Pafnucy, using the identical frame indices and the identical PLC-level train/test split. All other modeling choices (architecture, optimizer, hyperparameters; Table 4) were held fixed across every configuration, and only the frame-selection strategy and frame count were varied, mirroring the controlled design of the main Pafnucy study. The one procedural difference from the Pafnucy training protocol is that, for GIGN, a validation set comprising 10% of the training PLCs was carved out (at the PLC level) and used for early stopping and checkpoint selection. Pafnucy’s original training protocol did not use a held-out validation split. Aggregation across frames during inference (KDE, Mean, Bayesian GMM, FFNN) was performed in the same way as in the main text.

**Table 4:** GIGN hyperparameters used for the cross-architecture validation

Hyperparameter	Value
Node feature dimension	35
Hidden channel dimension	256
Pocket/non-covalent interaction distance threshold	5 Å
Optimizer	Adam
Learning rate	$5\times 10^{-4}$
Weight decay (L2)	$1\times 10^{-6}$
Gradient clipping (max norm)	5.0
Batch size (training)	128
Batch size (evaluation)	256
Maximum epochs	600
Early-stopping patience	100 epochs without validation improvement
Validation split	10% of training PLCs (split at the PLC level)
Loss function	MSE
Random seed	42

All settings were held fixed across every frame-selection strategy and frame count tested

### C.3: Results

The results are reported in Tables 5 and 6. Consistent with the main-text Pafnucy results, multi-frame training substantially outperforms the single-frame Baseline, and arithmetic Mean aggregation is the best or tied-best aggregation method in nearly every configuration, matching the robustness of simple averaging reported for Pafnucy. Notably, improvement over the baseline is sustained rather than tapering off: the best GIGN result is obtained at 60 frames using the clustered approach, the largest frame count we tested, rather than at an intermediate value, indicating that additional well-selected synthetic frames continued to help up to the range we evaluated.

**Table 5:** GIGN mean squared errors (in units of  $(\text {kcal} \cdot \text {mol}^{-1})^2$) obtained from the same analysis protocols as Pafnucy, evaluated on the training and testing datasets

Approach	Number of Frames	Train Set MSE					Test Set MSE				
		Direct	KDE	Mean	GMM	ANN	Direct	KDE	Mean	GMM	ANN
Baseline		0.07					**5.09**				
Uniform	10		0.05	0.05	0.05	0.06		4.63	4.44	4.48	4.46
	20		0.11	0.11	0.11	0.09		4.54	4.43	4.48	4.48
	30		0.20	0.20	0.20	0.22		4.63	4.53	4.63	4.50
	40		0.06	0.06	0.06	0.09		**4**.**44**	4.37	4.47	4.37
Clustered	10		0.08	0.08	0.08	0.11		4.49	4.37	4.42	4.40
	20		0.15	0.14	0.14	0.17		4.52	4.32	4.45	4.40
	30		0.09	0.08	0.08	0.10		4.45	**4**.**31**	**4**.**33**	**4**.**34**
	40		0.13	0.12	0.13	0.12		4.62	4.55	4.61	4.57
	50		0.17	0.17	0.17	0.17		4.61	4.41	4.54	4.48
	60		0.16	0.17	0.17	0.15		**4.10**	**3.98**	**4.10**	**4.11**

In each test-set column, the second-best-performing method is shown in bold, and the best is both bold and underlined

**Table 6:** GIGN Pearson correlation coefficients ($r_p$) of different methods evaluated on the training and testing datasets, similar to Pafnucy

Approach	Number of Frames	Train Set Pearson $r_p$					Test Set Pearson $r_p$				
		Direct	KDE	Mean	GMM	ANN	Direct	KDE	Mean	GMM	ANN
Baseline		1.00					**0.52**				
Uniform	10		1.00	1.00	1.00	1.00		0.57	0.59	0.59	0.59
	20		0.99	0.99	0.99	0.99		0.57	0.58	0.58	0.58
	30		0.99	0.99	0.99	0.99		0.58	0.58	0.57	0.58
	40		1.00	1.00	1.00	1.00		**0**.**59**	**0**.**60**	0.59	**0**.**60**
Clustered	10		1.00	1.00	1.00	0.99		**0**.**59**	**0**.**60**	0.59	**0**.**60**
	20		0.99	0.99	0.99	0.99		**0**.**59**	**0**.**60**	0.59	**0**.**60**
	30		0.99	0.99	0.99	0.99		**0**.**59**	**0**.**60**	**0**.**60**	**0**.**60**
	40		0.99	0.99	0.99	0.99		**0**.**59**	0.59	0.58	0.58
	50		0.99	0.99	0.99	0.99		0.58	0.59	0.58	0.59
	60		0.99	0.99	0.99	0.99		**0.62**	**0.63**	**0.62**	**0.63**

In each test-set column, the second-best-performing method is shown in bold, and the best is both bold and underlined

These results support the paper’s primary assertion by employing a structurally distinct, more recent architecture. They show that incorporating multiple MD-derived synthetic frames per PLC, chosen via clustering, meaningfully enhances binding-affinity prediction compared to a static-structure baseline, and that this advantage is not exclusive to Pafnucy. The exact frame count at which performance is maximized differs between the two architectures (30 for Pafnucy, 60—the upper edge of our tested range—for GIGN), which we note as a crucial fact that cautions against overgeneralizing a single optimal configuration across architectures.

We further note that GIGN exhibits a substantially larger train-test gap than Pafnucy, with training MSEs of 0.05-0.22  $(\text {kcal} \cdot \text {mol}^{-1})^2$ with $r_p = 0.99-1.00$, against test MSEs of 3.98-4.63 with $r_p = 0.57-0.63$ (Tables 5 and 6). This reflects the fact that GIGN’s hyperparameters (Table 4) follow the values reported by the original authors for PDBbind and were not re-tuned for the PLAS-20k dataset, and that GIGN has a larger effective capacity relative to our roughly 6000-PLC training set than the more heavily regularised Pafnucy (dropout 0.5, weight decay, 24-fold rotational augmentation; Table 3). Early stopping on the held-out validation split (Section C.2) limits but does not remove this effect. Because every frame-selection configuration is trained and evaluated under identical conditions, this gap does not affect the qualitative comparisons drawn here. However, the absolute GIGN errors should not be read as the best achievable performance for this architecture on PLAS-20k.

## Appendix D: Train-test sequence similarity and stratified robustness of the augmentation benefit

### D.1: Sequence-identity analysis

We quantified the protein-level similarity between the training and test sets. Our splits are performed at the level of PLCs: no complex appears in both sets. However, because the PLCs are drawn from PDBBind, in which the same protein frequently appears in complex with several different ligands, complex-level separation does not imply protein-level separation. To assess residual similarity, a single representative sequence was extracted from each PLC. For every test protein, the maximum pairwise sequence identity to any training protein was determined via local (Smith-Waterman) alignment, where identity was defined as the count of identical residues divided by the shorter sequence’s length. The mean of these per-protein maximum identities is 92.7%, and 77.5% of test proteins have an identical-sequence counterpart in the training set, confirming that the dataset is substantially redundant at the protein level, as is characteristic of PDBBind-derived data.

### D.2: Augmentation benefit within identity strata

This redundancy raises a natural question: is the improvement from multi-frame augmentation a genuine effect, or does it reflect the model recognizing proteins it has already seen? We therefore partitioned the test proteins into identity strata (< 30%, 30–50%, 50–90%, 90-<100%, and =100%) and, within each stratum, compared the single-frame baseline against the multi-frame (clustered) model, with per-PLC predictions aggregated by arithmetic mean. Because our concern here is whether the qualitative benefit is present in each stratum rather than its exact magnitude, we report the Pearson correlation ($r_p$) and the Spearman rank correlation (ρ) metrics between predicted and experimental affinities. Together, they capture both value-level and ranking-level agreement, the latter of which is especially relevant to virtual screening. Figure 4 reports both metrics, for the baseline and multi-frame models, at three frame counts spanning the range we tested (20, 40 and 60 frames, clustered selection).

Two observations hold consistently. First, in every stratum and at every frame count, both correlations increase substantially under multi-frame training relative to the baseline. This includes the low-identity (< 30%) stratum of typically structurally novel proteins, where the Pearson correlation rises from 0.29 for the baseline to 0.70–0.83 for the multi-frame models and the Spearman correlation from 0.15 to 0.58–0.70. Second, the improvement is of comparable magnitude between the novel proteins and the near-identical ones. We note that the 30–50% and 50–90% strata contain relatively few test proteins (n=18 and n=19, respectively), so the values in these two buckets are correspondingly noisier. It is the consistency of the trend across all five strata and across three independent frame counts, rather than any single bucket, that supports the conclusion.

### D.3: Interpretation

These results indicate that the improvement from multi-frame MD augmentation is preserved across the full range of train-test sequence similarity, including for proteins with little similarity to the training set. Therefore, it is not an artifact of the protein-level redundancy in the dataset. We emphasize that this analysis supports the qualitative conclusions of the paper, that augmentation with well-selected MD frames improves prediction, rather than the absolute error values, which are influenced by the dataset’s redundancy and should not be read as estimates of performance on entirely novel targets. Establishing the latter would require a protein-clustered split, which we identify as a direction for future work.

## Code availability

The underlying source code for this study is available at https://github.com/devalab/PL-Affinity-PLAS-20k.
